# Effects of External Employment Opportunities, Nursing Professionalism, and Nursing Work Environments on Korean Hospital Nurses’ Intent to Stay or Leave

**DOI:** 10.3390/ijerph20054026

**Published:** 2023-02-24

**Authors:** Mi-Aie Lee, So-Hee Lim

**Affiliations:** 1 Nursing Department, Nursing School, Dongguk University, Gyeongji-si 38066, Republic of Korea; 2 Department of Nursing, Kyungmin University, Uijeongbu-si 11618, Republic of Korea

**Keywords:** employment opportunity, intention, nursing work environment, nurses, Korea

## Abstract

This study aims to explore Korean Hospital nurses’ intent to stay or leave their working environment, and to identify the difference between the intent to stay and the intent to leave by identifying the relationship between external employment opportunities, professionalism, and work environment. Data were collected via an online survey and analyzed using stepwise multiple regression analysis. As a result of the analysis, the intent to stay among Korean hospital nurses was influenced by the work environment, external employment opportunities, education level, and marital status, whereas the intent to leave was influenced by the nursing work environment, marital status, and total clinical experience. As a result, the reflected variables differed. Thus, it can be concluded that hospital nurses’ intent to either stay or leave are not concepts that simply contradict each other in the same context but are, in fact, influenced differently by various factors. Nevertheless, it can also be concluded that nursing managers should make efforts to improve the nursing work environment to lower nurses’ intent to leave and increase their intent to stay by improving only the nursing work environment.

## 1. Introduction

Due to recent environmental changes, such as the population structure, disease patterns, emergence of infectious diseases, lifestyles, and social institutions, the social demand for nursing personnel has been steadily increasing. According to the Health and Medical Workforce Survey 2020, only 50.2% of licensed nurses in Korea work as hospital nurses, so the number of hospital nurses is 3.5 per 1000, which is less than half of the Organization for Economic Cooperation and Development average of 7.2 [1,2]. In addition, the turnover rate among nurses in Korea is 15.2%, which is almost eight times higher than the average turnover rate of 2.16% for other health and welfare workers [2]. However, with the inclusion of the intent to leave within 6 months, this rate increases by up to 31% [2]. This rate is more than twice as high as the claim by Toffler [3] that a turnover rate of 5–7% is appropriate for nurses. This indicates a serious issue in the healthcare domain, resulting in a shortage of Korean hospital nurses.

A high turnover is not necessarily disadvantageous. However, an excessive turnover among nurses must be managed, as it can threaten patient safety and incur costs for recruitment, selection, and training of new nurses to replace those who leave [4]. Moreover, this increases turnover, due to lower morale among the remaining nurses [5]. Most researchers who have conducted studies on the turnover among nurses have measured their intent to stay instead of the actual turnover [6]. This is because the intent to stay (or leave), although not being the same as turnover, could potentially explain the phenomenon of turnover [5,6]. 

The intent to leave refers to the behavior of a member of an organization leaving that organization or looking for a new job to relocate to. Conversely, the intent to stay refers to a member of an organization continuing with their job with the intent to remain at that organization [5]. In the past, the intention to stay or leave were regarded as opposing concepts [4]; however, they are now regarded as different concepts [5]. The intent to leave is induced by dissatisfaction owing to extrinsic and ancillary conditions, related to job performance and not being satisfied. This corresponds to the hygiene factors within Herzberg’s two-factor theory [6]. The intent to stay is induced by the work itself or personal achievement, responsibility, and recognition felt through work, which corresponds to the motivation factor within Herzberg’s two-factor theory [6]. Therefore, the management of the intent to leave passively contributes to maintaining nursing staff, whereas the management of the intent to stay actively contributes to maintaining nursing staff [4].

There are limited studies comparing and analyzing the factors affecting nurses’ intention to stay or leave between the same subjects in Korea [5]. Therefore, it is not possible to know exactly whether the intent to stay and the intent to leave are concepts with the same content or different concepts for hospital nurses. As a result, it is necessary to examine whether the two concepts have the same content or different content for hospital nurses. If the intention to stay or leave are actually the same concept, the same strategy can simply be applied in reverse to reduce the nurse turnover rate. However, if intent to stay or leave are different concepts, the strategy is used by focusing on the effective variable after evaluating to find out which one is effective for self-organization. Thus, it is important for nurses’ personnel management to confirm with hospital nurses whether intent to stay and intent to leave are the same or different concepts.

External employment opportunities represent the possibility of obtaining another job elsewhere [7]. According to a study by Mosadeghrad [8], 35% of nurses will leave their current hospital if given an external employment opportunity. Such claims are supported by studies that have reported that the intent to leave among Korean general hospital nurses is influenced by external employment opportunities [9]. Such opportunities are the main reason for high turnover among Korean hospital nurses, and account for 18.9% of the total reasons [1]. Therefore, having an external employment opportunity is understood to be a factor influencing the intent to leave among hospital nurses. However, the effects of an external employment opportunity on the intent to stay among hospital nurses could not be verified, as studies that identified the association between external employment opportunity and the intent to stay could not be located. Accordingly, the difference between the intent to stay or leave must be analyzed by identifying the effects of external employment opportunities on this issue among Korean hospital nurses. 

Nursing professionalism refers to the sum of the beliefs, ideas, and impressions that nurses have about nursing or the nursing profession [10]. Moreover, studies have reported that nursing professionalism can strengthen the intent to stay [11] by reducing role conflict and burnout among nurses [12]. It may, therefore, increase work performance [13] and lower turnover impulse and the intent to leave [13]. Many studies have examined the association between nursing professionalism and the intent to leave; however, few studies have examined the association between nursing professionalism and the intent to stay. Accordingly, the effects of nursing professionalism on both the intent to stay or leave among nurses must be identified. 

The nursing work environment is a concept that includes not only the physical environment required for nurses to perform their duties but also the interactions between nurses and other healthcare professionals and institutional policies [14]. This may promote work participation by nurses and enhance their work satisfaction to resolve the high turnover problem [15,16]. It has also been confirmed through studies [17,18] that the work environment is a factor, which allows nurses to remain in organizations. In addition, according to a previous study [5], it was found that work environment influenced nurses’ intent to leave, but not their intent to stay. Consequently, work environment may be assumed to have different effects on the intent to stay or leave among nurses. However, since this is the result of a previous study, the relationship between the nursing work environment and intent to stay or leave needs to be explored further.

Therefore, this study aims to confirm whether nurses’ intent to stay or leave are concepts with the same content or different concepts based on the turnover model of Price [7]. Accordingly, this study set ‘external employment opportunity’ as the environmental variable, ‘nursing professionalism’ as the personal variable, and the ‘nursing work environment’ as the structural variable in order to identify the effects of these three variables on the intent to stay or leave among Korean hospital nurses. In Price’s turnover model [7] intent to stay is an endogenous variable, but this study set intent to stay as the final variable for the purpose of comparing and analyzing the effect on the intent to stay or leave on the same participants.

Therefore, this study will enable Korean hospital nurses to know whether intent to stay or leave are the same or different concepts, so that nursing managers can select and manage effective variables to reduce the turnover rate of hospital nurses. It will provide basic data to solve the shortage of hospital nurses.

## 2. Materials and Methods

### 2.1. Study Design and Participants

The participants of this descriptive research were selected by convenience sampling and comprised nurses working at four general hospitals located in Seoul and the surrounding metropolitan area at the time of the study. Significantly, during the survey period, the number of confirmed COVID-19 cases had rapidly increased, and the hospitals had an infection control policy that prohibited contact with outsiders except for family members. Therefore, the metropolitan area was divided into four districts, and the survey was conducted at four hospitals based on convenience sampling. Nurses who were on a leave of absence were excluded. In addition, since the criteria for the intent to stay or leave in this study emphasized the roles and responsibilities of full-time nurses, nurses who worked more than 40 h per week were included. 

The questionnaires were administered between 15 July and 10 August 2021. The sample size was calculated using the G*Power 3.1.9.2 program with an effect size of 0.15. The significance level of 0.05, statistical power of 90%, and 22 predictors (11 general characteristics and 11 independent variables) based on a previous study on the intent to stay or leave among Korean hospital nurses [5] showed that the sample size needed for regression analysis was 198. Accordingly, 200 participants were recruited.

### 2.2. Measures

#### 2.2.1. External Employment Opportunities

External employment opportunity was measured by using a tool based on the questionnaire originally developed by Price and Mueller [19] and subsequently modified and supplemented by Oh, Seo, and Park [20] to be suitable for Korean hospital nurses. The tool consisted of three items graded on a 5-point Likert scale, with higher scores indicating higher external employment opportunities. The Cronbach’s α was 0.73 in the study by Oh et al. [20] and 0.82 in this study.

#### 2.2.2. Nursing Professionalism

Nursing professionalism was measured using the tool developed by Yeun, Kwon, and Ahn [10]. The tool consisted of 29 items in five subdomains (9 items on the self-concept of the profession, 8 items on social awareness, 5 items on the professionalism of nursing, 4 items on the roles of the nursing service, and 3 items on the originality of nursing). The items were graded on a 5-point Likert scale. Negative responses were reverse scored, and higher scores indicated higher nursing professionalism. The Cronbach’s α was 0.92 at the time of development by Yeun et al. [10] and 0.94 in this study.

#### 2.2.3. Nursing Work Environment

The nursing work environment was measured using the Practice Environment Scale of the Nursing Work Index (PES-NWI) originally developed by Lake [14] and subsequently translated and validated into the Korean version (K-PES-NWI) by Cho, Choi, Kim, Yoo, and Lee [21]. The tool consisted of 29 items in five subdomains (9 items on nurses’ participation in hospital affairs; 9 items on nursing foundations for quality of care; 4 items on nursing managers’ ability, leadership, and support of nurses; 4 items on staffing and resource adequacy; and 3 items on collegial nurse–physician relations). The items were graded on a 4-point Likert scale, with higher scores indicating a better nursing work environment. The Cronbach’s α was 0.93 in the study by Choi et al. [21] and 0.95 in this study.

#### 2.2.4. Intent to Stay or Leave

The intent to stay or leave was measured using a tool originally developed by Han and Lee [22] for Korean flight attendants and later modified by Lee et al. [5] to be suitable for Korean hospital nurses. The tool consisted of 16 items (8 items each for the intent to stay or leave that asked the same questions concerning the intent to stay or leave). The items were graded on a 5-point Likert scale, with higher scores indicating a higher intent to stay or leave. The study by Han and Lee [22] did not present the Cronbach’s α; however, the Cronbach’s α of the intent to stay or leave scales were 0.84 and 0.87, respectively, in the study by Lee et al. [5]; and 0.84 and 0.83, respectively, in this study. 

### 2.3. Ethical Considerations

Data were collected after obtaining approval from the Institutional Review Board of D University (no. 20210013). The first page of the online questionnaire contained information about the study. Participants were allowed to complete the questionnaire only after declaring their consent to participate in the study. 

### 2.4. Data Analysis

The collected data were computerized using SPSS/WIN 21.0 software and included frequency, percentage, mean, standard deviation, *t*-test, one-way analysis of variance followed by the Duncan test for post hoc analysis, Pearson’s correlation coefficient, and stepwise multiple regression analysis. Stepwise multiple regression analysis techniques are useful in identifying the major variables influencing the dependent variable and ranking them in order of importance [23].

## 3. Results

### 3.1. Participants’ General Characteristics

Most participants were women (n = 192; 96.0%). The mean age was 38.07 (±9.63) years, with the highest percentage of the participants being 40–49 years old (n = 62; 31.0%) followed by 30–39 and ≤29 years (n = 55; 27.5% each). Moreover, most participants were married (n = 108; 54.0%), had an education level of a master’s degree or higher (n = 109; 54.5%), and followed no religion (n = 93; 46.5%). Regarding the ward where they worked, the medical or surgical ward was the most common (n = 105, 52.5%), while the mean clinical experience was 12.39 ± 8.52 years, with most participants having 4 to 10 years of experience (n = 65; 32.5%). Regarding the size of the hospital where they worked, the most common response was a tertiary hospital with ≥800 beds (n = 83; 41.5%), while 110 participants (55.0%) responded that they had previous turnover experiences. The most common response for their average monthly income was 3,000,000–3,999,999 KRW (n = 78; 39.0%; Table 1).

### 3.2. Differences in the Intent to Stay or Leave according to Participants’ General Characteristics

The results showed the differences in the intent to stay according to age (F = 5.67; *p* = 0.001), marital status (t = 0.57; *p* < 0.001), education level (F = 13.21; *p* < 0.001), total clinical experience (F = 5.58; *p = 0*.001), and monthly income (F = 6.66; *p* < 0.001). The post hoc test results showed that nurses who are aged ≥50 years, married, and have a master’s degree or higher had a higher intent to stay than their peers. Additionally, nurses with a total clinical experience of ≥21 years and a monthly income of ≥4,000,000 won had a higher intent to stay than their peers (Table 1).

Furthermore, the results showed the differences in the intent to leave according to age (F = 9.34; *p* < 0.001), marital status (t = 3.80; *p* < 0.001), education level (F = 10.56; *p* < 0.001), total clinical experience (F = 8.44; *p* < 0.001), and monthly income (F = 8.11; *p* < 0.001). The post hoc test results showed that nurses who are aged ≤39 years, single, and have a bachelor’s degree or less had a higher intent to leave than their peers. Additionally, nurses with a total clinical experience of <21 years and a monthly income of ≤3,999,999 KRW had a higher intent to leave than their peers (Table 1).

### 3.3. Correlations among the External Employment Opportunities, Nursing Professionalism, Nursing Work Environment, and Intent to Stay or Leave

The intent to stay was correlated with external employment opportunities (r = −0.20; *p =* 0.005), nursing professionalism (r = 0.64; *p* < 0.001) and the nursing work environment (r = 0.57; *p* < 0.001), and the intent to leave was also correlated with external employment opportunities (r = 0.14; *p =* 0.043), nursing professionalism (r = −0.34; *p* < 0.001), and the nursing work environment (r = −0.47; *p =* 0.006). Moreover, the intent to stay or leave was also correlated (r = −0.60; *p* < 0.001; Table 2).

### 3.4. Influencing Factors of Intent to Stay or Leave 

The results showed a normal distribution with data appearing close to a 45-degree straight line, while partial scatter plots showed that the residuals were evenly distributed around the origin. The test of the autocorrelation of errors using the Durbin-Watson test were 2.14 and 2.06. As the values were larger than the test statistic value of 1.78, it was determined that there was no autocorrelation. Moreover, the tolerance limit index was ≥0.1 (0.82–0.98 and 0.86–0.98), and the variance inflation factor did not exceed the cutoff value of 10 (1.02–1.23 and 1.01–1.16).

The influencing factors of the intent to stay among the general characteristics were the nursing work environment (β = 0.54; *p* < 0.001) and external employment opportunities (β = −0.14; *p* = 0.001), along with an education level of a master’s degree or higher (β = 0.19; *p* = 0.002) and marital status (β = −0.17; *p* = 0.003). Among participants, these four variables had an explanatory power of 44% for the intent to stay. Furthermore, this regression equation was significant (F = 39.35; *p* < 0.001). 

Among the general characteristics, the factors influencing the intent to leave were the nursing work environment (β = −0.45; *p* < 0.001), the total period of clinical career (β = −0.18; *p* = 0.004), and marital status (β = 0.22; *p* < 0.001). These three variables had an explanatory power of 33% for participants’ intent to leave. In addition, this regression equation was significant (F = 33.13; *p* < 0.001; Table 3).

## 4. Discussion

Participants demonstrated a mean intent to stay score of 2.93 out of 5 possible points, which was slightly higher than the 2.58 points reported in a study that used the same tool to measure the intent to stay of regional general hospital nurses [5]. In Korea, regional small and medium-sized hospitals as well as large hospitals in the metropolitan area have different working conditions and welfare systems, and so there is a difference in nurses’ intent to stay [9,10]. Most participants were nurses working in general hospitals or higher-level medical institutions located in Seoul and the surrounding metropolitan area. Therefore, it is an expected result that their intent to stay is higher than that of nurses at regional general hospitals.

Participants demonstrated a mean intent to leave score of 2.89 out of five possible points, which was lower than the 2.99 points reported in a study that used the same tool to measure the intent to leave among regional general hospital nurses [5], and 3.26 points reported by a meta-analysis on the intent to leave among Korean hospital nurses [24]. According to one study [24], nurses who work in tertiary hospitals have a higher intent to leave than those who work in general hospitals. However, considering that 41.5% of this study’s participants worked in tertiary hospitals with ≥800 beds, their intent to leave was relatively low. Therefore, to confirm the results of this study, it is necessary to conduct repeated studies in future, targeting other hospital nurses working in different regions. According to this study and previous studies [5,9,11,12,13], Korean hospital nurses’ intent to stay or leave was found to be ‘normal’ at 3 points on a 5-point scale. This suggests that the turnover rate of hospital nurses can vary depending on how intent to stay or leave are managed. Therefore, nursing managers should make active efforts to increase nurses’ intent to stay at the hospital and lower their intent to leave.

Participants who were older, had longer total clinical experience, and a higher monthly income indicated a higher intent to stay. This finding is consistent with those of previous studies that have also reported differences in the intent to stay among nurses depending on personal factors, such as age, total clinical experience, and education level [5,11,25]. This may be attributed to an increased job capability and proficiency owing to the accumulation of clinical experience. It may also be attributed to increased job satisfaction due to increased job opportunities that allow nurses to feel a sense of achievement or allow them to work independently [16]. Therefore, methods to utilize experienced nurses must be explored to enhance the quality of care and resolve the problem of the nursing shortage. In this study, the participants who were older, had a longer total clinical experience, and were married demonstrated a lower intent to leave than their counterparts. This was consistent with the findings of previous studies [24,25,26] and indicated that experienced nurses have higher organizational loyalty than new nurses. Therefore, nursing managers can reduce turnover rates of hospital nurses as well as the quality of nursing by introducing policies such as career development systems, which provide different economic compensation or status according to career stage and use them to secure and retain experienced nurses.

The mean external employment opportunity score in this study was 3.33 out of 5 possible points. This differed from the 3.19 points reported in a study that used the same tool to measure the external employment opportunity among female nurses in tertiary hospitals [13]. Another study that measured external employment opportunities among male nurses reported 4.35 points [27]. The differences in nurses’ perceptions of external employment opportunities by sex are consistent with previous studies [13,27], suggesting that external employment opportunities can be perceived differently depending on the sex of nurses. This is a result of supporting the claim that nurses have gender stereotypes in Korea [27]. If the perception of external employment opportunities is different, the strategy for reducing the turnover rate should also be different. Therefore, in future, research is needed to explore the difference in perception of external employment opportunities according to the sex of nurses and the reason for this.

The mean nursing professionalism score in this study was 3.51 out of 5 possible points, which was similar to the 3.50 points reported in a study that used the same tool to measure nursing professionalism among general hospital nurses [11]. Nursing professionalism is positively correlated with organizational productivity variables, such as work performance [12], quality of care [13,24,25], and organizational competitiveness [11]. Therefore, it is necessary to continue to develop nursing professionalism among hospital nurses. Participants were nurses working in tertiary hospitals in Seoul and the surrounding metropolitan area. Considering that participants had a mean clinical experience of 12.39 years and 34.0% had a master’s degree or higher, it is highly likely that nursing professionalism had already been established in these participants. However, their nursing professionalism has yet to reach 4.0 with a score of 3.51, so further development is required. According to previous studies [12,13], nursing professionalism is developed through education and experience. Therefore, opportunities must be provided for continuing education for advancing to graduate schools or enhancing nursing professionalism. Furthermore, nursing professionalism must also be strengthened continuously among hospital nurses by expanding the direct nursing hours and opportunities to work independently, and by creating a mutually respectful organizational culture. Among the items within the category of nursing professionalism, social awareness had the lowest score in this study. Such findings indicate that despite the recognition of the social role and importance of nurses considering recent situations, such as the COVID-19 pandemic, compensation for and the social awareness of nurses are still not at a satisfactory level. There are limitations in attempting to improve the compensation for and social awareness of nurses through individual efforts. Therefore, individual nurses and nursing organizations must collaborate to improve the treatment and public image of nurses.

The mean nursing work environment score in this study was 2.49 out of 4 possible points. According to Lake [14], who developed the tool, such a score indicates that the nursing work environment is perceived as being mostly positive. Despite this, the mean scores for staffing and resource adequacy (2.26 points) and nurse participation in hospital affairs (2.36 points) were below 2.5 points. Therefore, hospitals and nursing managers should calculate the appropriate staffing for each institution based on the job analysis that accounts for the characteristics, conditions, and circumstances of each institution, and increases the support for material resources. In the process, material support for nurses and ways to participate in organizational management should be actively sought.

The analysis of correlations among the variables showed that the intent to stay was negatively correlated with external employment opportunities and positively correlated with nursing professionalism and the nursing work environments. The intent to leave was positively correlated with external employment opportunities and negatively correlated with nursing professionalism and the nursing work environments. These findings were consistent with the results of previous studies [5,24,25], indicating that the intent to stay increased with fewer external employment opportunities and better nursing professionalism and the nursing work environments. The intent to leave increased with more external employment opportunities and lower nursing professionalism. In this regard, nursing managers often find it difficult to control external employment opportunities; however, nursing professionalism and the nursing work environment can be enhanced. Therefore, nursing managers must increase their effort to increase nurses’ intent to stay and reduce their intent to leave by enhancing nursing professionalism and the nursing work environment. 

In this study, the factor with the highest influence on the intent to stay among Korean hospital nurses was the nursing work environment. This finding is consistent with previous studies on hospital nurses [5,15], indicating that the nursing work environment is important not only for nursing quality and positive outcomes but also for securing and retaining high quality nursing personnel [14]. The fact that the nursing work environment is important in reducing the turnover rate of nurses is consistent with the analysis of the intent to leave in this study. Furthermore, the factor with the largest influence on the intent to leave among Korean hospital nurses was identified to be the nursing work environment. This is also consistent with previous studies [5,16]. Dissatisfaction with the nursing work environment may increase intent to leave and impact staff turnover. In addition to lowering nurses’ morale and the need to manage the capabilities of the remaining nurses [5,15,16]. Therefore, nursing managers should make efforts to improve the nursing work environment, and ways to do this should include focusing on improving ‘appropriate manpower and material support’ and ‘nurse participation in hospital operations’.

Additionally, the factor with the second highest influence on intent to stay was identified to be external employment opportunities, which is an environmental factor. While controlling external employment opportunities is difficult for institutions, they can reduce the interest in external employment opportunities among employees by improving working conditions and creating a positive organizational culture to increase the level of work satisfaction and organizational commitment. However, since these assumptions are only conjectures, the relationship between hospital nurses’ intention to stay or leave and external employment opportunities needs a more in-depth analysis.

General characteristics of the subjects influencing intent to stay were marital status (married) and education level (master’s degree or higher), a result similar to that of a previous study [24]. It is thus assumed that married nurses have a higher intent to stay in the organization by pursuing security and stability compared to unmarried nurses. In addition, nurses with a high level of education will have a higher intent to stay in the department because of the opportunity to exercise expertise or discretionary increases as their position within the institution rises. Therefore, it is possible to increase a nurse’s intent to stay by utilizing strategies to support nurses’ studies along with strategies to support married nurses, such as parental leave or in-hospital childcare. 

Conversely, general characteristics of the subjects in this study that affect a hospital nurse’s intent to leave were marital status and total period of their clinical career, which are consistent with results of previous studies [5,24,25,26,27] targeting hospital nurses. Compared to married nurses, unmarried nurses are presumed to have higher intent to leave. Nurses with 1 to 3 years of clinical experience have more opportunities (with better conditions) to change jobs. As a result, to reduce the turnover rate of nurses, nursing managers need to recognize that not only new nurses with less than 1 year of clinical experience but also experienced nurses with less than 3 years of clinical experience are targets of active management, as the turnover rate is high [2]. In other words, married nurses with long careers and high educational levels had higher intent to stay and lower intent to leave than unmarried nurses with short careers and lower educational levels. Therefore, nursing managers can secure nurses with long clinical careers and reduce turnover rates by supporting nurses to work whilst studying or raising children.

The COVID-19 pandemic, which has been ongoing since 2019, has brought with it many changes. In particular, the work of hospital nurses has increased because of hospital closures owing to COVID-19 confirmed cases, cohort isolation and infection control, changes in real-time infection guidelines, and increased quarantine work. Additionally, they have been highly occupied with taking care of patients infected with COVID-19 for an extended period. This has resulted in severe burnout, physical discomfort, fear of infection, and psychological distress [28,29,30,31]. Significantly, the change in the working environment owing to high-intensity quarantine work and heavy work owing to COVID-19 has reportedly affected the turnover intention of nurses at Korean hospitals [28,31,32,33]. In this study, the factors influencing both the intent to stay or leave were supported by the nursing work environment. Therefore, the nursing work environment was an important variable that affected nurses’ intent to stay or leave even in rapidly changing situations or crises. Conversely, factors such as nurses’ service and a spirit of sacrifice made it possible to maintain a job despite the threat and anxiety caused by COVID-19 [30], and motivated nurses to stay in the hospitals. Such work motives reportedly reduced employee turnover even in times of crisis or difficulties related to the COVID-19 pandemic, such as salary, job security, and good teamwork, which reportedly corresponded to the motivations of employees to stay [34,35]. Among the general characteristics, the higher a nurse’s monthly income, clinical experience, age, and education level, the higher the intent to stay and the lower the intent to leave. In future, it is recommended that a program to strengthen the individual factors among nurses that affect their work motive be developed.

There are limitations in generalizing this study’s findings as the participants consisted solely of nurses working in Seoul and the surrounding metropolitan area. However, this study may contribute to the advancement of nursing theories by providing the basic data for developing theories related to the intention to stay or leave among nurses. In addition, this study confirmed the importance of the nursing work environment in increasing nurses’ intent to stay and lowering their intent to leave. Nursing organizations or managers should therefore actively improve the nursing work environment and contribute to the development of nursing practice. In addition, this study suggests that there is a difference in the perception of external employment opportunities according to the sex of hospital nurses, and thus it is recommended that future studies be conducted to confirm this.

## 5. Conclusions and Future Research

The results showed that the intent to stay among Korean hospital nurses was influenced by the nursing work environment, external employment opportunities, education level, and marital status. The intent to leave was influenced by the nursing work environment, marital status, and total clinical experience. Accordingly, the intent to stay and the intent to leave among Korean hospital nurses were concluded to be different concepts with different relations and variables. Therefore, nursing managers can resolve the shortage of nurses by selecting variables that their organizations can control to increase nurses’ intent to stay or reduce their intent to leave. The nursing work environment was a common factor that influenced both the intent to stay and intent to leave among hospital nurses. Therefore, it can be concluded that nursing managers can reduce the turnover rate of hospital nurses by specifically investigating the content of the nursing work environment to determine where their organization is vulnerable and then managing it more closely. In addition, it is necessary to develop a career development system to improve the quality and productivity of nursing care. It can also be concluded that a gender-specific management strategy is needed to reduce the external employment opportunities.

Based on the above conclusion, the following suggestions are made. First, replication studies are recommended to measure, compare, and directly analyze the intent to stay or leave among hospital nurses from different hospital sizes and areas. Second, some previous studies reported the influence of nursing professionalism on a nurse’s intention to stay or leave, but this study did not show any influence. Therefore, it is suggested to repeat a study to confirm the relationship between nursing professionalism and a nurse’s intention to stay or leave. Finally, policy development studies are recommended to develop strategies to improve the nursing work environment, which was identified as a common factor influencing both the intent to stay or leave among Korean hospital nurses.

## Figures and Tables

**Table 1:** Differences in the Intent to Stay or Leave based on Participants’ General Characteristics (n = 200).

General Characteristics		n (%)	Mean (±SD)	Intent to Stay		Duncan	Intent to Leave		Duncan
				Mean (±SD)	t/F(*p*)		Mean (±SD)	t/F (*p*)	
Sex	Male	8 (4.0)		2.83 ± 0.91	0.04 (0.714)		3.08 ± 0.99	0.87 (0.521)	
	Female	192 (96.0)		2.94 ± 0.81			2.88 ± 0.84		
Age (years)	≤29	55 (27.5)	38.07 ± 9.63	2.69 ± 0.69	5.67 (0.001)	b	3.14 ± 0.69	9.34 (<0.001)	a
	30–39	55 (27.5)		2.83 ± 0.74		b	3.13 ± 0.78		b
	40–49	62 (31.0)		3.02 ± 0.86		b	2.72 ± 0.86		c
	≥50	27 (14.0)		3.40 ± 0.85		a	2.31 ± 0.89		c
Marital status	Married	108 (54.0)		3.12 ± 0.80	0.57 (<0.001)		2.66 ± 0.86	3.80 (<0.001)	
	Single	92 (46.0)		2.71 ± 0.76			3.13 ± 0.74		
Education level	Diploma	23 (11.5)		2.72 ± 0.88	13.21 (<0.001)	b	2.85 ± 0.75	10.56 (<0.001)	ab
	Bachelor’s	109 (54.5)		2.74 ± 0.76		b	3.11 ± 0.75		a
	≥Master’s	68 (34.0)		3.32 ± 0.72		a	2.54 ± 0.91		b
Religion	Protestant	53 (26.5)		3.04 ± 0.72	2.44 (0.066)		2.84 ± 0.79	0.17 (0.914)	
	Buddhism	22 (11.0)		3.26 ± 0.99			2.82 ± 0.99		
	Catholic	32 (16.0)		2.93 ± 0.65			2.89 ± 0.87		
	None	93 (46.5)		2.79 ± 0.82			2.93 ± 0.81		
Working place	Medi/Surgi ward	105 (52.5)		2.99 ± 0.80	0.81 (0.448)		2.87 ± 0.87	0.12 (0.889)	
	ICU, ER, OR	43 (21.5)		2.80 ± 0.77			2.89 ± 0.84		
	OPD	52 (26.0)		2.93 ± 0.86			2.84 ± 0.81		
Total period of clinical career (years)	≤3	36 (18.0)	12.39 ± 8.52	2.64 ± 0.77	5.58 (0.001)	b	3.15 ± 0.68	8.44 (<0.001)	a
	4–10	65 (32.5)		2.84 ± 0.80		b	3.11 ± 0.83		a
	11–20	59 (29.5)		2.84 ± 0.76		b	2.83 ± 0.81		a
	≥21	40 (20.0)		3.34 ± 0.78		a	2.38 ± 0.83		b
Hospital size (beds)	<300	66 (33.0)		2.87 ± 0.84	0.30 (0.826)		2.84 ± 0.96	0.48 (0.695)	
	300–399	17 (8.5)		2.96 ± 0.80			2.79 ± 0.75		
	400–799	34 (17.0)		3.03 ± 0.75			3.03 ± 0.78		
	≥800	83 (41.5)		2.93 ± 0.82			2.90 ± 0.79		
Experiences of turnover	No	90 (45.0)		2.86 ± 0.83	0.75 (0.241)		2.84 ± 0.80	1.03 (0.498)	
	Yes	110 (55.0)		2.99 ± 0.79			2.93 ± 0.88		
Monthly income (10,000 won)	<200	2 (1.0)		2.13 ± 0.53	6.66 (<0.001)	b	3.63 ± 0.99	8.11 (<0.001)	a
	200–299	68 (34.0)		2.70 ± 0.81		ab	2.98 ± 0.78		ab
	300–399	78 (39.0)		2.91 ± 0.72		ab	3.10 ± 0.85		ab
	≥400	52 (26.0)		3.30 ± 0.81		a	2.44 ± 0.76		b

SD = standard deviation; ICU = intensive care unit; ER = emergency room; OR = operating room; OPD = out-patient department. a > ab > b > c the difference between the mean values of each group.

**Table 2:** Correlation among the Variables (n = 200).

Variables	External Employment Opportunity	Nursing Professionalism	Nursing Work Environment	Intent to Stay	Intent to Leave
	r(*p*)				
External employment opportunity	1				
Nursing professionalism	0.19 (<0.001)	1			
Nursing work environment	−0.02 (0.821)	0.47 (0.001)	1		
Intent to stay	−0.20 (0.005)	0.64 (<0.001)	0.57 (<0.001)	1	
Intent to leave	0.14 (0.043)	−0.34 (<0.001)	−0.47 (0.006)	−0.60 (<0.001)	1

**Table 3:** Stepwise Multiple Regression Analysis on the Intent to Stay or Leave.

Variable		B	SE	β	t	*p*
Intent to stay	Constant	1.37	0.26		5.20	<0.001
	Marital status	−0.28	0.09	−0.17	−2.96	0.003
	Education level (2)	0.32	0.10	0.19	3.14	0.002
	External employment opportunity	−0.12	0.05	−0.14	−2.56	0.011
	Nursing work environment	0.80	0.08	0.54	9.93	<0.001
R^2^ = 0.45, Adjusted R^2^ = 0.44, F = 39.35, *p* < 0.001, Durbin-Watson’s d = 2.14						
**Variables**		**B**	**SE**	**β**	**t**	***p***
Intent to leave	Constant	4.57	0.24		19.03	<0.001
	Marital status	0.38	00.11	0.22	3.56	<0.001
	Total period of clinical career	−0.39	.13	−0.18	−2.94	0.004
	Nursing work environment	−0.71	0.09	−0.45	−7.77	<0.001
R^2^ = 0.34, Adjusted R^2^ = 0.33, F = 33.13, *p* < 0.001, Durbin-Watson’s d = 2.06						

Dummy variables: marital status (married = 0, single = 1), education level (diploma = 0, bachelor’s = 1, master’s or higher = 2). SE = standard error.

## Data Availability

The data presented in this study are available on request from the corresponding author. The data are not publicly available owing to the information contained that could compromise the privacy of research participants.
